# Management and prognosis of patients with ovarian sex cord tumor with annular tubules: a retrospective study

**DOI:** 10.1186/s12885-015-1277-y

**Published:** 2015-04-12

**Authors:** Qiuhong Qian, Yan You, Jiaxin Yang, Dongyan Cao, Zhaohui Zhu, Ming Wu, Jie Chen, Jinghe Lang, Keng Shen

**Affiliations:** 1 Department of Obstetrics and Gynecology, Peking Union Medical College Hospital, Chinese Academy of Medical Sciences & Peking Union Medical College, No. 1 Shuaifuyuan, Dongcheng District, Beijing, 100730 P.R. China; 2 Department of Pathology, Peking Union Medical College Hospital, Chinese Academy of Medical Sciences & Peking Union Medical College, Beijing, P.R. China; 3 Department of Nuclear Medicine, Peking Union Medical College Hospital, Chinese Academy of Medical Sciences & Peking Union Medical College, Beijing, P.R. China

**Keywords:** Sex cord tumor with annular tubules, Treatment, Prognosis, Ovarian tumor, Peutz–Jeghers syndrome

## Abstract

**Background:**

Owing to the rarity of sex cord tumor with annular tubules (SCTAT), it is difficult to recognize SCTAT clinically and there is no standard treatment. The aim of our study was to investigate the treatment outcomes and prognosis of patients with ovarian SCTAT.

**Methods:**

A cohort of 13 patients with SCTAT diagnosed and treated in Peking Union Medical College Hospital was studied. Data on clinicopathological characteristics, treatment, and prognosis were retrospectively reviewed and analyzed.

**Results:**

SCTAT accounted for 1.4% of ovarian sex cord stromal tumors, with an average onset age of 22.6 years. All patients presented with menstrual disturbances or isosexual precocity at disease onset. Initial surgery was unilateral salpingo-oophorectomy in 11 cases. Recurrence rate was 46.2%, and 38.5% of patients experienced multiple recurrences. The disease free interval gradually shortened with increasing numbers of recurrences. Recurrent tumors were mostly ipsilateral to the primary tumor and located in retroperitoneum. Surgery remained the main treatment for recurrent cases. Serum estradiol and progesterone levels usually elevated at disease onset, decreased dramatically after operation, and they elevated again with the development of recurrence. The median progression-free survival (PFS) was 97.8 months, and the 1-year and 5-year PFS were 92% and 67%, respectively. Five-year overall survival (OS) was 100%.

**Conclusions:**

Unilateral salpingo-oophorectomy is a feasible treatment for primary SCTAT cases with intact capsules and without PJS. Complete tumor resection is suggested for recurrent cases and long-term follow-up is strongly recommended. Despite the high risk of recurrence, SCTAT prognosis is relatively favorable.

## Background

Sex cord tumor with annular tubules (SCTAT) is a rare and distinctive ovarian sex cord-stromal tumor (SCST), accounting for approximately 2.3% of SCSTs [1]. The predominant component of this tumor has intermediate morphologic features between granulosa cell tumors and Sertoli cell tumors and focal differentiation into either of these tumor types may occur [2]. In 1970, Scully first described this distinctive tumor [3], and a series of case reports have since been published. The most notable is a review of 74 SCTAT cases in 1982 [2]. They demonstrated clinical and pathological findings and their associations with Peutz–Jeghers syndrome (PJS). Among these cases, 36.5% were associated with PJS and tumors in these patients were usually benign, multifocal, bilateral, calcified, and very small or even microscopic in size. Tumors in patients without PJS (sporadic SCTAT) were unilateral, large, and 21.9% cases were clinically malignant [2]. However, most literature on SCTAT was case reports [4-11] and there exists a serious lack of reports with larger samples. Owing to its rarity, it is difficult to recognize SCTAT clinically, and there is no agreed**-**upon management. In the present study, we reported a cohort of 13 SCTAT cases and aimed to investigate the treatment outcomes and prognosis.

## Methods

### Study population

A total of 936 SCST cases were registered at Peking Union Medical College Hospital (PUMCH) from 1968 to 2014. Thirteen patients with SCTAT were diagnosed and treated at the same period (1.4%). These cases were retrospectively collected and reviewed by searching the ovarian tumor database. The study was approved by the ethics committee in Peking Union Medical College Hospital, Beijing, People’s Republic of China. Of the 13 patients with SCTAT, eight cases were treated for primary disease and five cases were referred from other hospitals because of recurrence after unilateral salpingo-oophorectomy. The following information was collected: age, parity, complaint, clinical features, imaging findings, tumor markers, sex hormone levels, treatment modalities, and previous treatment records. The diagnosis of SCTAT was confirmed by histopathology and reviewed by two independent pathologists. The slides of original ovarian tumors in patients with recurrence were reviewed by pathologists in our hospital.

### Treatment protocol

For patients with primary SCTAT, treatment in our hospital was mainly unilateral salpingo-oophorectomy. For patients with recurrent SCTAT, complete recurrent tumor resection (RTR) was the main treatment of choice. After surgical treatment, patients with large tumors (≥30 cm) or recurrences were treated with postoperative adjuvant chemotherapy. Three cycles of standard combination chemotherapy regimens (PEB—cisplatin 30–35 mg/m^2^, intravenous drip, day 1–3; VP16 100 mg/m^2^, intravenous drip, day1–3; and bleomycin 15 mg/m^2^, intramuscular, days 2, 9, and 16; or PVB—cisplatin 30–35 mg/m^2^, intravenous drip, day 1–3; vinblastin 1–1.5 mg/m^2^, intravenous drip, day 1–2; and bleomycin 15 mg/m^2^, intramuscular days 2, 9, and 16) were given as the main adjuvant chemotherapy. Chemotherapy was repeated every 3 weeks. PEB or PVB regimen with full dosage, adequate cycles, and strict adherence was considered as standard chemotherapy.

### Follow-up

All patients had regular follow-up after treatment, including clinical examination, tumor marker (e.g., sex hormones) measurements, and imaging tests at least every 3 months during the first year following treatment and at gradually increasing intervals thereafter. Follow-up duration was defined from the day of initial surgery to the last visit or death. Disease recurrence was defined if clinical and/or imaging examinations demonstrated visible disease or histopathology-confirmed diagnosis of SCTAT after a documented disease-free interval following initial therapy or after a documented complete response to therapy. Progression-free survival (PFS) was defined as the time from the date of initial surgery to the date of disease progression or recurrence. Overall survival (OS) was defined as the time from the date of initial surgery to the date of death or last visit.

### Statistical analysis

Data from the present study were analyzed using standard descriptive statistics (e.g., frequencies and percentages). Comparisons of frequency distribution between categorical variables were made using the Chi-square test or Fisher’s exact test. Life table was used for survival analysis. Two sided *P*-values < 0.05 were considered statistically significant. Statistical analysis was conducted with SPSS 17.0 software (IBM Corp, Armonk, NY, USA).

## Results

### Clinical features

**Figure 1:**
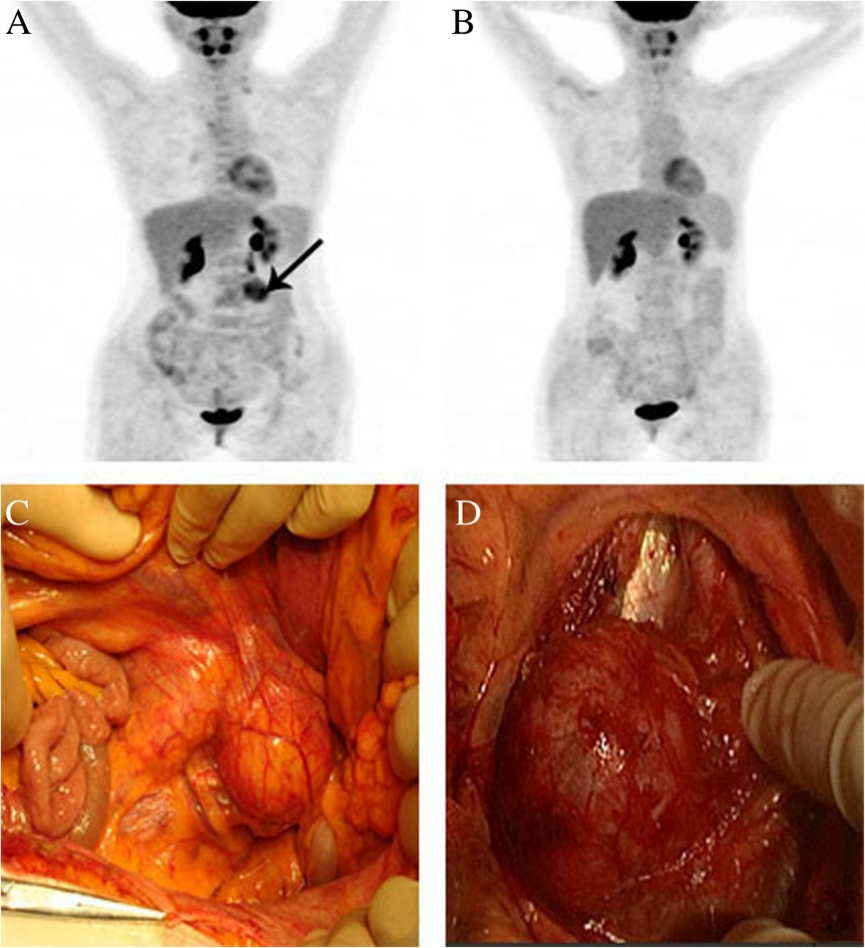
PET-CT scan and macroscopic findings in a recurrent patient (case 11). (**A**) PET-CT scan before treatment; a black arrow points to the metastatic tumor in the left portion of the fourth lumbar vertebra. (**B**) PET-CT scan after treatment. (**C**) and (**D**) show the retroperitoneal tumor fused by several para-aortic lymph nodes.

Thirteen patients with SCTAT were identified from 936 SCST cases admitted in PUMCH at the same period, accounting for 1.4% of SCST cases. Clinicopathologic features, treatment modalities, and follow-up data are presented in Table 1. The average onset age of the 13 patients was 22.6 ± 12.1 years old (range 5–39 years) and 6 (46.2%) patients were ≤18 years old. All patients had menstrual disturbances at disease onset, including seven patients presenting with amenorrhea, three patients presenting with prolonged menstrual bleeding, and three patients presenting with isosexual precocity (breast budding, pubic hair growth, and early menarche before the age of 8 years old) at the age of 8, 6, and 5 years old, respectively. Besides, before the presentation of amenorrhea, three patients had irregular menstrual cycles and four patients had irregular menstrual bleeding with prolonged menses. Patients with recurrent tumors also presented with menstrual disturbances. Abdominal or pelvic masses were found in all patients by physical examinations and/or with imaging tests such as ultrasonography, computed tomography (CT), or positron emission tomography (PET)-CT scan (Figure 1A).

**Table 1:** **Clinicopathologic features, treatment modalities, and follow-up data of SCTAT cases**

No.	Age		Previous treatment	Side of tumor: size	Treatment		Recurreces	Treatment for subsequent recurrence	Follow-up	
	Onset	Admission			Surgery	Chemo			Month	Outcome
1	18	26	^a^RSO	Right:-	^a^TAH + LSO + RTR + LN biopsy	PVB*3	2	-	146	DOD
2	31	34	^a^LSO	Left:7 cm	^a^TAH + RSO + omentum + LND + RTR+ RPL	5-Fu*1,N-formylsarcolysin *5,TSPA*2	3	Chemo: PVB*4; ^a^RTR + RPL + RT	179	PR
3	15	15	No	Right:30 cm	^a^RSO	nitrosourea	0	-	84	CR
4	8	8	No	Left:9 cm	^a^LSO + biopsy of right ovary	-	0	-	48	CR
5	31	31	No	right:13 cm;left:microscopic	^a^RSO and biopsy of left ovary	-	0	-	24	CR
6	21	29	^a^RSO	Right:24 cm	^a^TAH + LSO + RTR + omentum	PVB*2	3	^a^RTR;RT	143	PR
7	18	20	^a^LSO; RTR + RPL + RT + Chemo	Left:20 cm	^a^RTR + RPL + LND	PEB*3	2	-	26	CR
8	39	39	No	Right:5 cm	^b^RSO and biopsy of left ovary	-	0	-	55	CR
9	6	6	^a^Biopsy of ovary	Left:8 cm	^a^LSO	-	0	-	63	CR
10	39	39	No	Right:12 cm	^a^TAH + BSO + LND + omentum	PEB*3	0	-	107	CR
11	29	30	^a^LSO	Left:6 cm	^a^RTR + RPL + LND	PEB*3	1	-	21	CR
12	34	34	No	Right:12^+^cm	^b^Cystectomy	-	3	^a^RSO + RTR + omentum + appendix; RTR^a^ + chemo; RTR^a^	51	PR
13	5	5	No	Left:8 cm	^b^Biopsy of ovary; ^b^LSO	-	0	-	20	CR

Note: Chemo, chemotherapy; RSO, right salpingo-oophorectomy; TAH, total abdominal hysterectomy; LSO, left salpingo-oophorectomy; RTR, recurrent tumor resection; LN, lymph nodes; RPL, retroperitoneal lymphadenectomy; DOD, died of disease; 5-Fu, 5-fluorouracil; TSPA, Thiotepa; PVB, cisplatin + vinblastine + bleomycin; RT, radiotherapy; PR, partial remission; CR, complete remission; PEB, cisplatin + etoposide + bleomycin; BSO, bilateral salpingo-oophorectomy; LND, lymph node dissection; ^a^laparotomy; ^b^laparoscopy.

Of the 13 patients with SCTAT, eight cases were treated for primary tumors and five cases were referred from other hospitals for recurrent tumors. One case (case 5) had pigmentation of the oral mucosa, but had no documentation of gastrointestinal polyps. None of the other patients had documented PJS presentations. In eight cases with primary tumors, only two pediatric patients (case 9, 13) had a preoperative diagnosis of SCTAT based on biopsy of ovarian tumor. In the five recurrent cases referred from other hospitals, only case 11 was diagnosed with SCTAT histopathologically at the referring hospitals.

Table 2 compares the clinicopathologic features and treatment modalities between patients with and without recurrence. Patients with age < 18 years old, bilateral ovarian tumors, postoperative chemotherapy, or cytoreductive surgery (CRS) did not experience recurrence during follow-up. However, no statistically significant difference was found in clinicopathologic features and treatment modalities between patients with and without recurrence (*P* > 0.05).

**Table 2:** **Comparison of clinicopathologic features and treatment modalities between patients with and without recurrence (Chi-square test)**

Parameters	Recurrence		P value^a^
	Yes ( 6 cases)	No (7 cases)	
age			
≥18 years	6 (66.7%)	3 (33.3%)	0.07
<18 years	0 (0%)	4 (100%)	
Side of tumor			
left	3 (50%)	3 (50%)	0.52
right	3 (50%)	3 (50%)	
bilateral	0 (0%)	1 (100%)	
Chemotherapy^b^			
Yes	0 (0%)	2 (100%)	0.46
no	6 (54.5%)	5 (45.5%)	
surgery			
USO	5 (45.5%)	6 (54.5%)	0.25
Cystectomy	1 (100%)	0 (0%)	
CRS	0 (0%)	1 (100%)	
Ovarian tumor size			
>10 cm	2 (40%)	3 (60%)	1.00
≤10 cm	4 (50%)	4 (50%)	

Note: USO, unilateral salpingo-oophorectomy; CRS, cytoreductive surgery, predominantly including total hysterectomy, bilateral salpingo-oophorectomy, omentectomy and pelvic lymphadenectomy; ^a^Fisher’s exact test. ^b^refers to adjuvant chemotherapy after initial surgical treatment.

### Treatment

Of the eight patients with primary tumors, six (75%) underwent unilateral salpingo-oophorectomy and biopsy of the contralateral ovary was performed in three of them (case 4, 5, 8). The other two patients were treated respectively with laparoscopic ovarian cystectomy (case 12) and CRS (case 10; including total hysterectomy, bilateral salpingo-oophorectomy, omentectomy, and bilateral pelvic lymphadenectomy) because a SCTAT diagnosis with the possibility of malignancy was made by intraoperative frozen section examination. Pelvic lymph nodes were negative in case 10. Another five cases referred from other hospitals underwent unilateral salpingo-oophorectomy previously as initial therapy in the referring hospitals.

There were a total of 14 recurrences in six recurrent patients. RTR (Figure 1C and D) was performed for five recurrences in four patients. CRS including total hysterectomy, contralateral salpingo-oophorectomy, and RTR was performed in three patients. CRS with fertility preservation was performed for three recurrences in one patient (case 12). Four patients underwent lymphadenectomy, of whom two underwent ipsilateral pelvic and para-aortic lymphadenectomy (case 2,11), case 7 underwent bilateral pelvic and para-aortic lymphadenectomy and case 1 underwent pelvic lymph node biopsy. Positive pelvic lymph nodes were found in three patients (case 1, 2, 7) and positive para-aortic lymph nodes were found in three patients (case 2, 7, 11). After surgical treatment, all recurrent patients were treated by adjuvant chemotherapy and two patients were treated by radiotherapy owing to supraclavicular lymph node metastasis. Chemotherapy or radiotherapy alone was performed for recurrences in two patients, respectively.

In patients with primary tumors, only two patients were treated by surgery combined with chemotherapy, case 3 for a large tumor (30 cm in diameter; nitrosourea) and case 10 for a SCTAT diagnosis with the possibility of malignancy (three cycles of PEB). For recurrent patients, PEB or PVB was the main chemotherapy regimen and was administered in five patients (case 1, 2, 6, 7, 11). One patient (case12) with multiple recurrences and extensive metastasis accepted chemotherapy regimens including PV (cisplatin + vinblastine), TC (paclitaxel + carboplatin), and PT (cisplatin + paclitaxel).

### Histopathological characteristics

**Figure 2:**
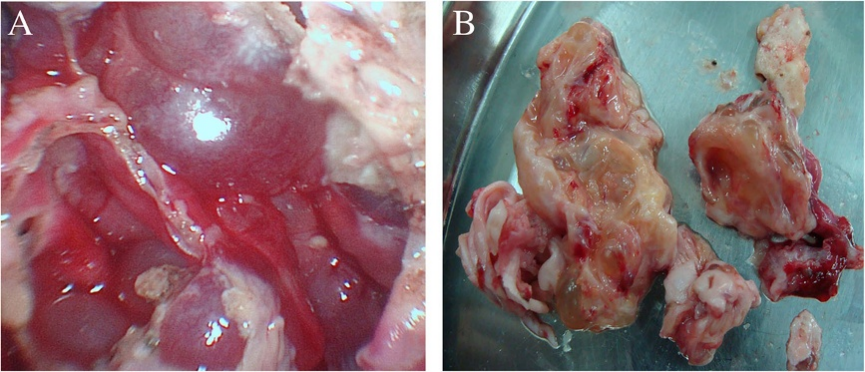
Macroscopic findings in a 5-year-old patient (case 13). (**A**) Multilocular cystic tumor ranging in size from 0.5 to 1.0 cm inside the cyst. (**B**) The sectioned surface of the tumor showed clear yellow fluid and multiple yellow nodules inside the cyst wall.

All primary tumors were confined to ovaries and had intact capsules, with an average diameter of 12.1 cm (range 5.0–30.0 cm). Tumors originated from the left ovary in six cases, right ovary in six cases, and bilateral ovaries in only one case (case 5) with a microscopic tumor in the left ovary and a 13 cm tumor in the right ovary. All the pediatric patients (<10 years old) had polycystic tumors filled with clear yellow fluid and associated with multiple yellow nodules ranging in size from 0.5 to 1.0 cm inside the cyst (Figure 2). Tumors in other patients including recurrent cases had yellow section surfaces.

Recurrent tumors were mostly ipsilateral to the primary tumor and located mainly in retroperitoneum, such as pelvic lymph nodes, para-aortic lymph nodes, and other sites in retroperitonium. Besides, supraclavicular lymph node metastasis was found in three patients (case 2, 6, 11). Extensive metastasis in the abdominal and pelvic cavity was found in two cases (case 7, 11) and recurrent tumors were also mainly ipsilateral to the primary tumor. No metastasis was found in the uterus and during the long-term follow-up no metastasis was found in the contralateral ovary.

**Figure 3:**
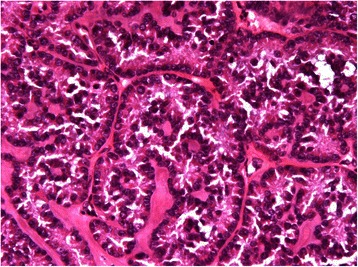
Pathologic findings in case 10 (H&E staining × 400 magnification). The tumor shows circumscribed columnar epithelial nests and multiple tubules encircling hyalinized basement membrane-like material.

The typical SCTAT structure is characterized by circumscribed columnar epithelial nests composed of ring-shaped tubules, which are encircled by hyalinized basement membrane-like material (Figure 3) [12]. No calcification was seen in tumor tissues. Mitotic count was only reported in two patients (case10, 12), and varied from 1 to 3 per 10 high power fields (HPF). Histopathological diagnosis of endometrium was made in seven patients, including three cases of glandular atrophy with decidual change in the stroma, three cases of proliferative endometrium, and one case of secretory endometrium with decidual change in the stroma.

Immunohistochemical staining was made in seven cases; a 100% (7/7) positive rate was seen for alpha-inhibin, 80% (4/5) for calretinin, 66.7% (2/3) for CD99, 50% (1/2) for Melan-A, and 66.7% (2/3) for AE1/AE3. Tumors were negative for Chromogranin A (CgA; 0/3), synaptophysin (Syn; 0/3), epithelial membrane antigen (EMA; 0/4), p53 (0/2), S-100 (0/2), CD10 (0/1), Wilms’ tumor gene 1 (WT-1; 0/1), and placental alkaline phosphatase (PLAP; 0/1). Proliferation-associated antigen Ki67 ranged from 2% to 30%.

### Tumor markers and sex hormones

Serum CA125 levels were tested in seven cases and only case 12 had abnormal CA125 levels after the second recurrence with a maximum level of 211 U/mL. No abnormality was found in other tumor markers, including CEA (0/4), AFP (0/6), CA199 (0/4), CA242 (0/2), CA153 (0/2), and CA724 (0/1).

**Figure 4:**
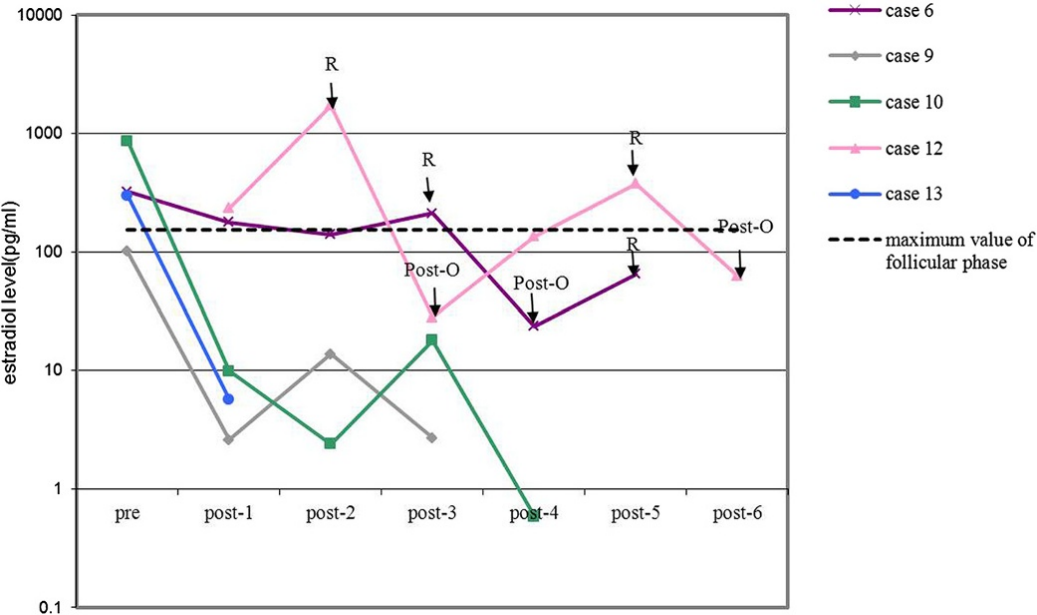
Preoperative and post-operative estradiol levels in five patients with SCTAT. Pre = preoperative test of estradiol; post-n = nth test of estradiol after initial treatment in our hospital; R = recurrence; Post-O = post-operation after recurrence.

**Figure 5:**
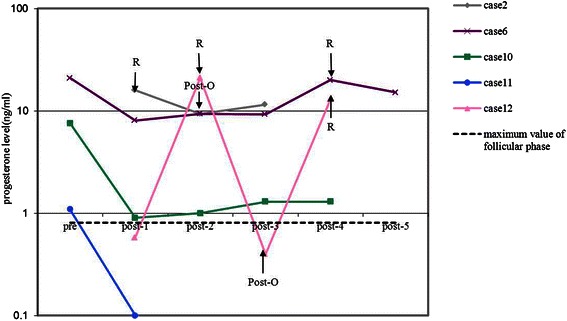
Preoperative and post-operative progesterone levels in five patients with SCTAT. Pre = preoperative test of progesterone; post-n = nth test of progesterone after initial treatment in our hospital; R = recurrence; Post-O = post-operation after recurrence.

Levels of estradiol (Figure 4) and progesterone (Figure 5) were only monitored dynamically in five patients, respectively. Both estradiol and progesterone levels decreased markedly when tumors were removed and elevated dramatically with the presence of recurrence. Levels of testosterone were monitored dynamically in six patients. However, only one patient (case 10) had elevated testosterone level (64 ng/dL) at disease onset and decreased to 5.4 ng/dL after surgical treatment. Other hormone levels, such as prolactin, luteinizing hormone (LH), or follicle stimulating hormone (FSH), did not show obvious tendencies.

### Follow-up

During follow-up, six (46.2%) patients experienced recurrence. There were a total of 14 recurrences in these six patients. Multiple recurrences (range 2–3) were observed in five (38.5%) patients. Mean duration from initial surgical treatment to first recurrence, from first recurrence to second recurrence, and from second recurrence to third recurrence were 45.5 months (range 7–108 months), 29.2 months (range 5–77 months), and 18.7 months (range 12–30 months), respectively. The most notable case was one patient (case 12) who developed three recurrences and extensive metastasis in the abdominal and pelvic cavity, including omentum, diaphragm, peritoneum, retroperitoneum, and abdominal wall at 9, 19, and 31 months, respectively, after laparoscopic ovarian cystectomy in our hospital. Eventually she achieved partial remission (PR) and was alive with disease after three fertility-sparing CRS surgeries.

Mean follow-up duration was 74.5 months (range 20–179 months). The median PFS was 97.8 months, and the 1-year and 5-year PFS were 92% and 67%, respectively. The 5-year OS was 100%. In our study, seven patients with primary disease achieved complete remission (CR) and were alive without recurrence after surgical treatment and two recurrent patients also achieved CR without recurrence after RTR combined with PEB or PVB chemotherapy. Three recurrent patients achieved PR and were alive with disease after surgery and/or radiotherapy. Case 1 died of progressive disease and cachexia after two recurrences 12 years after treatment.

## Discussion

SCTAT is a rare SCST accounting for only 1.4% of SCSTs in our study. SCTAT diagnosis is usually based on pathological examination of the tumor and typical clinical presentations are usually consistent with a hormone-secreting tumor, resulting in precocious puberty in children or menstrual disturbances, such as amenorrhea, in reproductive age women. Some researchers have demonstrated that SCTAT secretes not only estradiol, but also progesterone [12-14]. Our study also found endometrial decidual change in the stroma consistent with the progesterone effect in four patients. However, preoperative or even intraoperative SCTAT diagnosis is rather difficult. In our study, preoperative SCTAT diagnosis was only made in two pediatric patients based on ovarian tumor biopsy, both of whom were highly suspected of physiological cysts instead of ovarian tumors. Thus, biopsy was chosen to reduce the damage to ovarian tissue. However, this results in disruption of the capsule and may change prognosis of ovarian tumors. It turns out to be prudent to perform biopsy in patients with ovarian mass.

There is no standard treatment protocol for patients with SCTAT because of its rarity. Initial management for patients with SCTAT is surgery. It is very important to preserve fertility because most cases occur in adolescents or in reproductive age women. In our study, the median onset age was 22.6 years, which is similar to a previous report [2]. Tumors in SCTAT patients without PJS are usually unilateral and most patients can be diagnosed at early stages owing to hormone-related symptoms. Thus, fertility-sparing surgery is feasible for those young patients. However, ovarian cystectomy is not suggested because of the high rate of recurrence. In our study, only one patient was treated with ovarian cystectomy and experienced three recurrences and extensive metastasis in the abdominopelvic cavity within 31 months after initial surgery. Unilateral salpingo-oophorectomy is feasible for patients with an intact capsule and tumor confined to one ovary. In our study, six patients achieved CR without recurrence after treatment with unilateral salpingo-oophorectomy. Wedge section or biopsy of the contralateral ovary is not a routine procedure, but it should be considered, especially for patients with suspected lesion in the contralateral ovary. Because patients with PJS usually have bilateral ovarian tumors [2], it is prudent to maintain fertility for patients with any manifestation of PJS, such as hamartomatous polyps of the gastrointestinal tract, mucocutaneous melanin pigmentation, and various neoplasms. Even if the tumor is confined to one ovary, wedge section or biopsy of the contralateral ovary is strongly recommended for patients with PJS. Moreover, in our study, no metastasis was found in the contralateral ovary or uterus during follow-up. For patients without fertility desires, the effects of contralateral ovary resection and/or hysterectomy on prognosis should be further investigated.

Different from epithelial ovarian cancer, surgery is still the main treatment of choice for patients with recurrent SCTAT. Recurrent tumors tend to be retroperitoneal and can be detected and located by imaging tests, such as ultrasonography, CT, or PET-CT scan (Figure 1A). Preoperative evaluation is very important for recurrent SCTAT. PET-CT scan is a favorable method for the evaluation of tumor size, numbers, location of recurrent tumors, and the therapeutic effect (Figure 1A and B). Fertility-sparing surgery can be still considered for patients with no involvement of contralateral ovary and uterus. In our study, fertility-sparing surgery with complete RTR was performed in three patients. Patients with multiple recurrences can also achieve CR or PR after RTR or CRS.

Because the majority of recurrent tumors located in retroperitoneum, particularly in pelvic lymph nodes ipsilateral to the primary tumor and/or para-aortic lymph nodes, ipsilateral pelvic and para-aortic lymphadenectomy may be considered to reduce recurrence. In our study, only one patient (case 10) underwent bilateral pelvic lymphadenectomy as part of initial treatment and achieved CR afterwards. Two recurrent patients who underwent ipsilateral pelvic and para-aortic lymphadenectomy achieved CR (case 11) and PR (case 2), respectively. To date, there are only a few reports of lymphadenectomy or lymph node biopsy in patients with SCTAT and they lacked long-term follow-up data [7,9,15]. The role of lymphadenectomy, especially ipsilateral pelvic and para-aortic lymphadenectomy, in the treatment of SCTAT patients should be further investigated.

The effects of chemotherapy and radiotherapy are not clear. Although four patients (case 3, 10, 7, 11) achieved CR after surgery combined with chemotherapy and two patients (case 2, 6) achieved PR after radiotherapy with/without surgery in our study, chemotherapy or radiotherapy cannot completely protect patients from recurrence. Further studies are needed to confirm the effects of chemotherapy and radiotherapy.

SCTAT was thought to be an ovarian tumor with low malignant potential and late recurrence [12]. 21.9% of cases were clinically malignant in patients with sporadic SCTAT [2], and only few PJS-associated SCTAT cases with malignant behavior have been reported [7]. In our study, recurrence rate reached 46.2% and multiple recurrences were observed in 38.5% of patients, which indicated the malignant potential of SCTAT. Although the recurrence rate is high, the prognosis of patients with SCTAT is relatively favorable. Most recurrences were controlled well by surgeries and/or adjuvant therapy, and the 1-year and 5-year PFS were 92% and 67%, respectively. The median PFS time was 97.8 months, but the disease free interval gradually shortened with increasing numbers of recurrences. The 5-year OS was 100%.

Although SCTAT prognosis is relatively favorable, the risk of recurrence is still very high. Long-term follow-up is essential for patients with SCTAT. Our results demonstrated that recurrent patients would present with menstrual disturbances and pelvic or abdominal masses again at the time of recurrence. Thus, careful history taking and physical examination combined with imaging tests can be used as monitoring methods. In addition, both serum estradiol and progesterone can be used as tumor markers during follow-up. Elevated serum inhibin level was also reported in patients with SCTAT by several researchers [9,16] and immunohistochemical staining in our patients demonstrated that the most common marker was alpha-inhibin, with a positive rate of 100% (7/7). Thus, serum inhibin may also be used for follow-up.

## Conclusions

Unilateral salpingo-oophorectomy is feasible for primary patients with intact capsules and without PJS. Complete tumor resection is recommended for recurrent cases and fertility-sparing surgery can be considered for recurrent patients with no involvement of the contralateral ovary and uterus. Despite the high risk of recurrence, SCTAT prognosis is relatively favorable. Long-term follow-up is strongly recommended. Further studies are needed to confirm the effects of pelvic and para-aortic lymphadenectomy, chemotherapy, and radiotherapy in the treatment of patients with SCTAT.
